# Nuclear Translocation of β-Catenin during Mesenchymal Stem Cells Differentiation into Hepatocytes Is Associated with a Tumoral Phenotype

**DOI:** 10.1371/journal.pone.0034656

**Published:** 2012-04-10

**Authors:** Carmen Herencia, Julio M. Martínez-Moreno, Concepción Herrera, Fernando Corrales, Raquel Santiago-Mora, Isabel Espejo, Monserrat Barco, Yolanda Almadén, Manuel de la Mata, Antonio Rodríguez-Ariza, Juan R. Muñoz-Castañeda

**Affiliations:** 1 Maimónides Institute for Biomedical Research (IMIBIC)/Reina Sofia University Hospital/University of Córdoba, Córdoba, Spain; 2 Cellular Therapy Unit, IMIBIC/Reina Sofia University Hospital, Córdoba, Spain; 3 Center for Applied Medical Research, University of Navarra, Proteomics Laboratory, Pamplona, Spain; 4 Service of Clinic Analysis, Reina Sofía University Hospital, Córdoba, Spain; 5 Liver Research Unit, CIBERehd, IMIBIC/Reina Sofia University Hospital, Córdoba, Spain; Northwestern University Feinberg School of Medicine, United States of America

## Abstract

Wnt/β-catenin pathway controls biochemical processes related to cell differentiation. In committed cells the alteration of this pathway has been associated with tumors as hepatocellular carcinoma or hepatoblastoma. The present study evaluated the role of Wnt/β-catenin activation during human mesenchymal stem cells differentiation into hepatocytes. The differentiation to hepatocytes was achieved by the addition of two different conditioned media. In one of them, β-catenin nuclear translocation, up-regulation of genes related to the Wnt/β-catenin pathway, such as Lrp5 and Fzd3, as well as the oncogenes *c-myc* and *p53* were observed. While in the other protocol there was a Wnt/β-catenin inactivation. Hepatocytes with nuclear translocation of β-catenin also had abnormal cellular proliferation, and expressed membrane proteins involved in hepatocellular carcinoma, metastatic behavior and cancer stem cells. Further, these cells had also increased auto-renewal capability as shown in spheroids formation assay. Comparison of both differentiation protocols by 2D-DIGE proteomic analysis revealed differential expression of 11 proteins with altered expression in hepatocellular carcinoma. Cathepsin B and D, adenine phosphoribosyltransferase, triosephosphate isomerase, inorganic pyrophosphatase, peptidyl-prolyl cis-trans isomerase A or lactate dehydrogenase β-chain were up-regulated only with the protocol associated with Wnt signaling activation while other proteins involved in tumor suppression, such as transgelin or tropomyosin β-chain were down-regulated in this protocol. In conclusion, our results suggest that activation of the Wnt/β-catenin pathway during human mesenchymal stem cells differentiation into hepatocytes is associated with a tumoral phenotype.

## Introduction

Wnt/β-catenin signaling pathway is a master regulator of cell fate and proliferation during embryonic development that plays a main role in the control of differentiation of embryonic and adult stem cells [1]. A key element of this pathway is β-catenin, a multifunctional protein with important functions in intracellular adhesion, cell growth, survival and differentiation [2]. In the canonical Wnt/β-catenin pathway, nuclear β-catenin is associated with T cell factors and lymphoid enhancer-binding factor1 leading to transcriptional activation of target genes that regulate many cellular processes, such as cell cycle control through *c-myc* or cellular differentiation [3]. Wnt/β-catenin is activated during mesenchymal stem cells (MSC) differentiation to osteoblasts [4] and inactivated during differentiation into adipocytes [5]. Some studies have also recently documented the inactivation of this pathway during differentiation of MSC into hepatocytes [6], [7], [8]. The aberrant activation of Wnt/β-catenin pathway in committed cells has been related with the development of several types of tumors, including hepatocellular carcinoma or hepatoblastoma [9], [10], [11]. The Wnt/β-catenin pathway could be implicated in the mechanisms that participate in the progression of functional differentiated hepatocytes into tumor cells [10], [11], [12], [13].

Stem cells and cancer are inextricable linked and emerging data suggest an association between alterations in stem cells and the generation of cancer stem cells (CSC) [14], [15], [16]. However, the mechanisms by which stem cells adopt CSC properties are presently unknown. In this context it is particularly interesting to study the consequences of the activation of Wnt/β-catenin pathway during MSC differentiation into hepatocytes and its relationship with the occurrence of a tumoral phenotype. This study examines the effects of Wnt/β-catenin activation during the differentiation of MSC into hepatocytes as well as on the association of Wnt/β-catenin pathway activation with the generation of a tumoral phenotype.

## Results

### Immunophenotype of human mesenchymal stem cells before and after differentiation into hepatocytes

**Figure 1:**
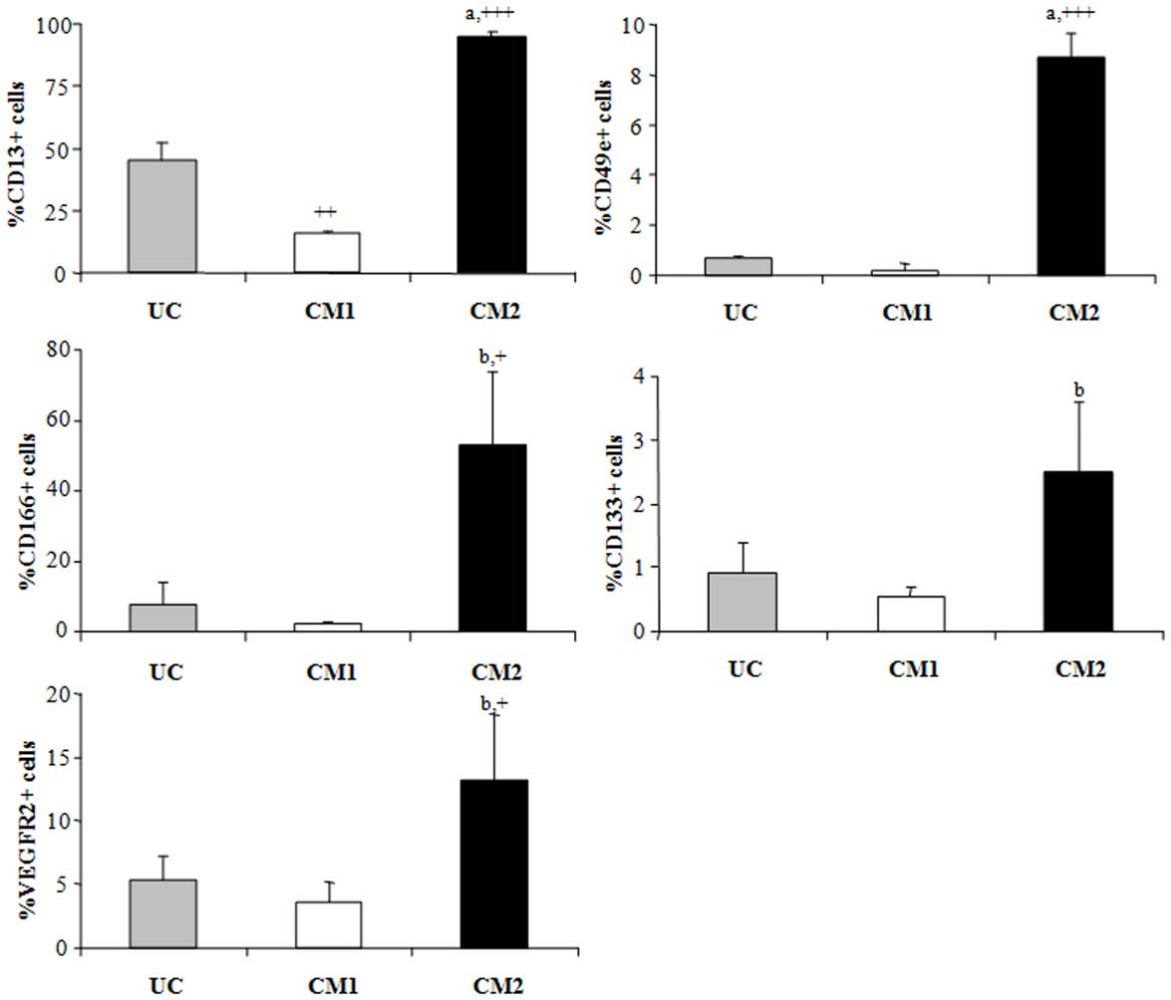
Differential stem cells markers in undifferentiated and differentiated human mesenchymal stem cells. Levels of CD13, CD49e, CD166, CD133 and VEGFR2 in undifferentiated cells (UC), CM1 and CM2-treated cells after 21 days of culture. (Conditioned medium: CM). Values are expressed as mean of percentage ± standard deviation. (a p<0.001 and b p<0.01 vs. CM1-treated cells; +++ p<0,001 and + p<0,05 vs. undifferentiated cells).

Human MSCs specific markers were evaluated by flow cytometry before and after 21 days of treatment with two protocols (CM1 and CM2) of hepatocytes differentiation. At 0 days, undifferentiated human bone marrow MSCs were negative for the expression of CD34, CD45, CD117, CD133, CD184 and VEGFR2, but positive for the expression of CD13, CD26, CD29, CD44, CD49e, CD90, CD105 and CD166 (Table 1). When human MSCs were cultured during 21 days for hepatocytes differentiation, marked differences in the levels of CD13, CD49e, CD133, CD166 and VEGFR2 were observed in CM1 and CM2-treated cells. The levels of these markers were significant higher in CM2-treated cells as compared to undifferentiated cells or CM1-treated cells (Figure 1).

**Table 1:** Stem cells/cancer stem cells markers expression in undifferentiated cells at time 0 days.

	Undifferentiatedcells (t = 0)
**% CD13**	99.5±0.04
**% CD29**	99.8±0.11
**% CD34**	2.02±0.158
**% CD44**	99.5±0.45
**% CD45**	1.18±1.491
**% CD49e**	61.8±13.09
**% CD73**	99.5±0.40
**% CD90**	99.6±0.13
**% CD105**	98.8±0.52
**% CD133**	1.35±0.979
**% CD166**	67.9±16.58
**% CXCR4**	0.96±0.179
**% VEGFR2**	2.04±0.311

Data are included as mean ± standard deviation.

### Expression of hepatospecific markers in human mesenchymal stem cells during their differentiation into hepatocytes

**Figure 2:**
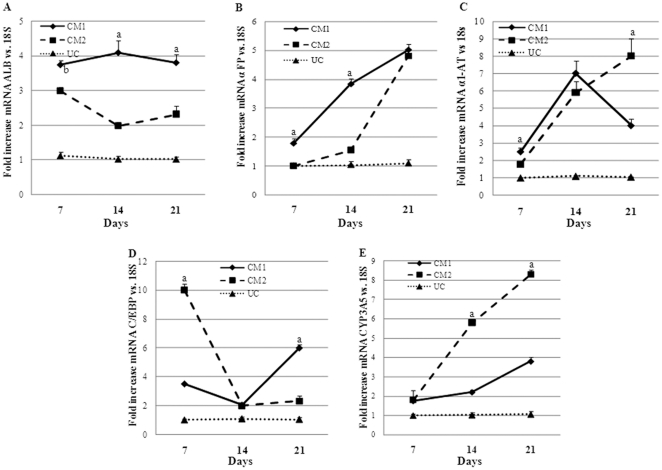
The treatments with CM1 or CM2 increase the expression of hepatospecific genes in human mesenchymal stem cells. Relative levels of mRNA expression of A) albumin (ALB), B) α-fetoprotein (αFP), C) α1-antitrypsin (α-1-AT), D) CCAAT/enhancer-binding protein beta (C/EBP) and E) cytochrome P450 (CYP3A5) were determined in human undifferentiated mesenchymal stem cells before and after differentiation with conditioned medium 1 (CM1) or 2 (CM2) after 7, 14 and 21 days of culture; Gene expression is shown as fold-changes compared to undifferentiated cells at each time. Values are expressed as mean ± standard deviation. All genes were increased significantly respect to undifferentiated cells (UC). ^a^ p<0.001, ^b^ p<0.01 vs. CM1 or CM2.

To compare the potential for hepatic differentiation of both protocols, the expression of hepatospecific genes was evaluated measuring mRNA levels by RT-PCR and protein expression by immunocytochemistry. The mRNA levels of albumin (ALB), α-fetoprotein (αFP), α1-antitrypsin (α1-AT), C/EBPα and CYP3A5 were strongly induced in human MSCs treated with CM1 or CM2 at 7, 14 and 21 days, compared to undifferentiated cells (UC). Comparatively, at 21 days of differentiation, there are no significant differences between both differentiation protocols. In CM2-treated cells there were not significant differences in the expression of these genes between cells to time 0 and cells after 48 h of treatment. The expressions of albumin and C/EBP were bigger in CM1 than CM2-treated cells while the expressions of α1-AT and CYP3A5 were increased in CM2 vs. CM1-tretaed cells. The expression of αFP was similar with both protocols (Figure 2).

**Figure 3:**
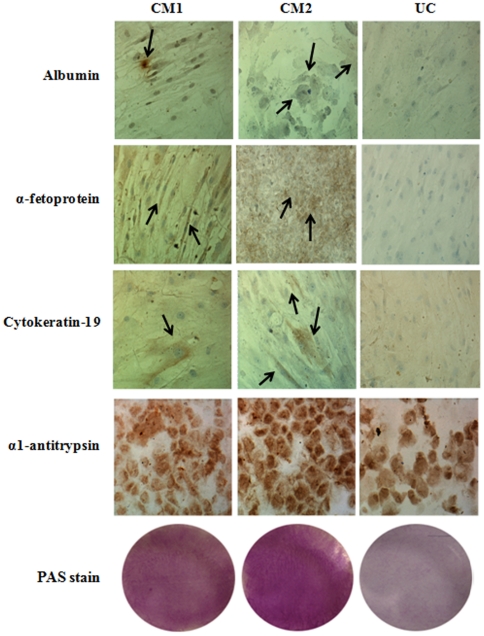
The treatment with CM1 or CM2 induces the presence of hepatospecific proteins in human mesenchymal stem cells. The presence of hepatospecific proteins such as albumin, α 1-antitrypsin, α-fetoprotein, cytokeratin-19 and PAS stain were evaluated by immunohistochemistry after 21 days of culture with conditioned medium CM1 or CM2. Arrows show positive staining area.

Expression of hepatospecific proteins, such as albumin, α1-antitrypsin, α-fetoprotein and cytokeratin 19 were also immunodetected in cells after 21 days of differentiation with both protocols CM1 or CM2 (Figure 3). Both protocols expressed with similar intensity hepatospecific proteins. Undifferentiated cells at 21 days were negative for the expression of these proteins. PAS stain was positive after treatment with CM1 and CM2 although a higher intensity was observed in cells cultured with CM2.

### Role of Wnt/β-catenin pathway and p53 during human mesenchymal stem cells differentiation into hepatocytes

**Figure 4:**
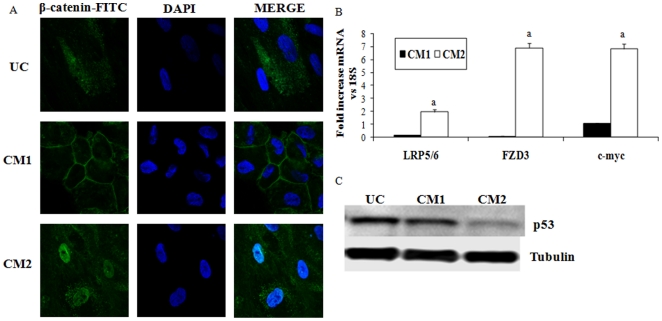
The treatment of human mesenchymal stem cells with CM2 induces nuclear translocation ofβ-catenin and Wnt signaling activation. A) To determine β-catenin subcellular localization, human mesenchymal stem cells undifferentiated (UC) and treated with conditioned medium 1 (CM1) or 2 (CM2) after 21 days of culture were stained for β-catenin immunofluorescence (green) and counterstained with DAPI (blue). Merged image of β-catenin-FITC and DAPI staining is also shown. Original magnification: 40×. B) mRNA expression of Lrp5/6, Frizzled- 3 (FZD3) and c-myc was evaluated in undifferentiated cells and cells treated with conditioned medium 1 (CM1) or 2 (CM2). Fold of undifferentiated cells at 21 days of culture. ^a^ p<0.001 vs. CM1-treated cells. C) Figure 4 c shows western blot of p53 and α-tubulin as loading control. Image is representative of three independent experiments.

Since Wnt/β-catenin pathway plays a main role in the control of differentiation of adult stem cells, confocal microscopy was used to study the subcellular localization of β-catenin during differentiation into hepatocytes in both CM1 and CM2 (Figure 4A). Immunofluorescence staining demonstrated that β-catenin localized to the cell membrane or to the peri-membrane region in undifferentiated and CM1 cells after 21 days, while there was no evidence of nuclear translocation. In contrast, there was a clear localization of β-catenin to the nuclei of differentiated human MSCs after treatment with CM2.

To confirm Wnt/β-catenin pathway activation during CM2 protocol, the expression of several genes regulated by this pathway, such as Lrp5/6, Frizzled 3 and c-myc, were next analyzed. The differentiation of human MSCs into hepatocytes with CM2 increased the mRNA expression of Lrp5, Frizzled 3 and c-myc. Conversely, undifferentiated cells and CM1-treated cells showed much lower levels of expression of these genes (Figure 4B).

Figure 4c shows western blot of p53 and tubulin as loading control. The expression of p53 was similar in undifferentiated and CM1-treated cells however its expression was significantly reduced in CM2-treated cells.

### Wnt/β-catenin activation leads to abnormal proliferation and spheroids formation

**Figure 5:**
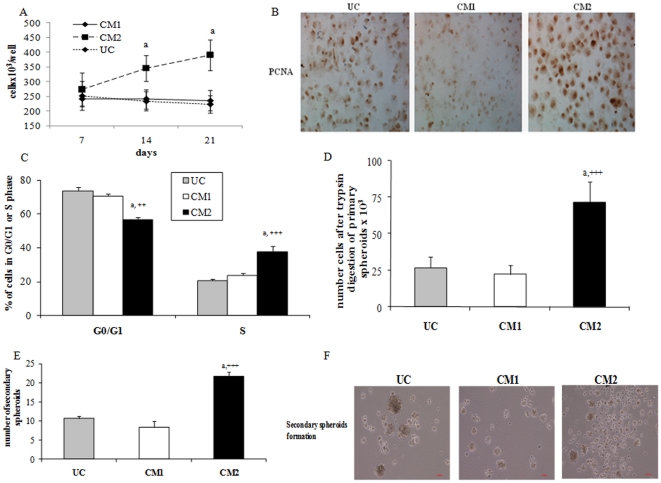
Markers of tumoral phenotype. A) Number of cells after 7, 14 and 21 days of culture in undifferentiated, CM1 and CM2-treated cells. Values expressed as mean ± standard deviation. a p<0.001 vs. CM1-treated cells and undifferentiated cells. B) The presence of nuclear PCNA (brown nucleus) was evaluated by immunohistochemistry after 21 days of culture in undifferentiated cells, CM and CM2-treated cells. Image is representative of three experiments. Original magnification: 20×. C) Cell cycle was analyzed at 21 days of hepatocyte differentiation in undifferentiated, CM1 and CM2-tretaed cells. Data are showed as mean of percentage plus standard deviation. a p<0.001 vs. CM1-treated cells, ++ p<0.01 and +++ p<0.001 vs. undifferentiated cells. D) Primary spheroid assay with count of number of cells after 4 days of culture with conditioned medium for spheroid formation. Data are showed that mean ± standard deviation (a p<0.001 vs. CM1-treated cells and +++ p<0.001 vs. undifferentiated cells). E) Secondary spheroid formation assay. Number of secondary spheroids was counted in an inverted microscope. Three experiments were carried out and data are expressed as mean ± standard deviation (a p<0.001 vs. CM1-treated cells and +++ p<0.001 vs. undifferentiated cells). F) Detail of secondary spheroids is showed in the microphotographs of undifferentiated cells, CM1 and CM2-treated cells.

Figure 5a shows that after 14 days of hepatocytes differentiation the number of CM2-treated cells begins to be higher with this treatment than CM1-treated cells or undifferentiated cells. At 21 days of hepatocytes differentiation, in CM2-treated cells there was a 75% more of cells than in undifferentiated or CM1-treated cells (a p<0.001 vs. CM1-treated cells and undifferentiated cells at 14 days and 21 days).

Nuclear staining of PCNA was significantly higher in CM2-treated cells than in undifferentiated or CM1-treated cells (Figure 5b). PCNA staining reinforces the abnormal proliferation detected in CM2-treated cells. With respect to cell cycle, Figure 5c shows a similar percentage of cells in G0/G1, G2/M and S phase in undifferentiated cells and CM1-treated cells. However in CM2-treated cells it is interesting to note a significant increase in the percentage of cells in S phase as well as a decrease in G0/G1 phase with respect to undifferentiated and CM1-treated cells.

For spheroid assay, differentiated cells for 21 days were cultured in low adherent plates for 4 days. Primary spheroids were detected in all groups although the number of spheres seemed be higher in CM2-treated cells. To quantify this data spheres were digested with trypsin-EDTA and subsequently counted. It is interesting to note that the capability to form spheres and the number of cells was higher in CM2-treated cells than the other cells (Figure 5d). After 4 days more of culture in low adherence plates and a clonal dilution the number of secondary spheres was significantly higher in CM2-treated cells than in undifferentiated cells (+++ p<0.001) and CM1-treated cells (a p<0.001). There was not difference in the number of spheres between undifferentiated and CM1-treated cells (Figure 5e). A detail of these secondary spheroids is showed in the microphotographs of Figure 5f. 3D structure of spheroids is showed in the movie of Supporting Information files (Figure S1). 3D animation of detected spheres was observed in each treatment; however we show only an example of this 3D-structure in this case a spheroid corresponding to CM2-treated cells.

### Analysis of protein expression profile by DIGE

**Figure 6:**
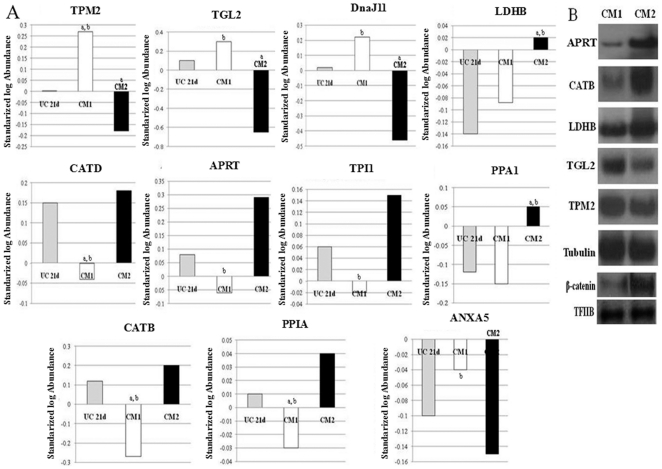
The activation of Wnt/β-catenin during hepatocyte differentiation is associated with the presence of related proteins to tumoral phenotype. Relative abundance of specific proteins (DIGE analysis) in human mesenchymal stem cells undifferentiated after 21 days of culture (UC21d) and in mesenchymal stem cells differentiated into hepatocytes with conditioned medium 1 (CM1) or 2 (CM2). B) Western blot confirmation of the changes observed by DIGE analysis in the abundance of some proteins in CM1 and CM2 hepatocytes: Adenine phosphoriobosyl transferase (APT), cathepsin B precursor (CATB), L-lactate dehydrogenase β chain (LDHB), transgelin (TGL2), tropomyosin β chain (TPM2) and nuclear β-catenin. Tubulin and TFIIB were used as cytoplasm and nuclear loading control respectively.

A proteomic DIGE approach was used to analyze the repertoire of proteins differentially expressed in control cells and hepatocytes obtained with CM1 or CM2 differentiation protocols. The DIGE analysis showed 39 differentially expressed proteins, and 17 of them were identified, including chaperones, metabolic, structural, proteolytic and apoptosis-related proteins (Table 2). Eleven of these proteins were differentially expressed in CM1 vs. CM2 (Figure 6). The differential expression in CM1 vs. CM2 of proteins, such as adenine phosphoribosyl transferase, transgelin, cathepsine B precursor, tropomyosin β chain and L-lactate dehydrogenase β chain was confirmed by western blots (Figure 5B). DIGE analysis showed a higher expression of adenine phosphoribosyltransferase, cathepsin B and D, triosephosphate isomerase, inorganic pyrophosphatase, peptidyl-prolyl cis-trans isomerase A or L-lactate dehydrogenase β-chain in hepatocytes obtained after treatment with CM2, than in CM1-treated or undifferentiated cells. In contrast, the expression of other proteins, such as transgelin, tropomyosin β chain, annexin A5 or Dna J homologous subfamily B decreased in hepatocytes obtained after treatment with CM2, compared to CM1-treated or undifferentiated cells. Nuclear β-catenin was also more expressed after treatment with CM2 than in CM1-treated cells.

**Table 2:** Comparative analysis by DIGE of proteins differentially expressed in hepatocytes obtained with CM1 or CM2 differentiation protocols.

Protein name	NCBI Acc n°	Sequenced Peptides	Sequence Cov (%)	Av. Ratio CM1/CM2	p value	Subcellular location	Molecular function
**Structural proteins**							
Tropomyosin beta chain	P07951	10	33	−2,68	0,0031	Cyt	Muscle contraction
Transgelin	Q01995	5	32	−9,61	0,0024	Cyt/Nuc	Muscle protein
Collagen alpha-2 (VI) chain	P12110	3	2,9	-	-	Secr	Extracellular matrix structural constituent
**Chaperones**							
Heat-shock protein beta-1	P04792	7	40	-	-	Cyt/Nuc	Heat shock protein binding
DnaJ homolog subfamily B member 11 precursor	Q9UBS4	1	17	−4,82	0,0015	ER	Heat shock protein binding
Peptidyl-prolyl cis-trans isomerase A	P62937	2	17	-	-	Cyt	Isomerase
**Metabolic enzymes**							
Triosephosphate isomerase	P60174	11	56	1,45	0,0260	Cyt	Isomerase
Inorganic pyrophosphatase	Q15181	2	14	1,57	0,0270	Cyt	Inorganic diphosphatase activity
Adenine phosphoribosyltransferase	P07741	4	25	1,89	0,0027	Cyt	AMP binding
L-lactate dehydrogenase beta chain	P07195	5	29	−9,64	0,0026	Cyt	Oxidoreductase
NADH ubiquinone oxidoreductase 30 KDa subunit	O75489	1	9	-	-	Mit	NADH dehydrogenase (ubiquinone) activity
Glutamate dehydrogenase 1 mitochondrial	P00367	1	8	-	-	Mit	Oxidoreductase
**Apoptosis-related proteins**							
Peroxirredoxin-4	Q13162	4	16	-	-	Cyt	Thioredoxin peroxidase activity
Elongation factor 1-delta	P29692	4	24	-	-	Cyt	Signal transducer activity
Annexin A5	P08758	22	73	-	-	Cyt	Calcium-dependent phospholipid binding
**Proteases**							
Cathepsin B precursor	P07858	4	18	2,79	0,0140	Lys	Hydrolase, protease, thiol proteose
Cathepsin D precursor	P07339	7	29	1,57	0,0410	Lys	Aspartyl protease, hydrolase

Cyt: Cytoplasm; Nuc: Nucleus; Secr: Secreted protein; ER: Endoplasmic reticulum; Mit: Mitochondria; Lys: Lysosome.

## Discussion

Hepatocytes differentiation has been achieved using different types of stem cells, MSC [17], embryonic stem cells [18] or induced pluripotent stem cells [19]. However in these studies the role of Wnt/β-catenin activation during hepatogenesis is unclear. In our study, we used human MSC and two different protocols to achieve differentiation into hepatocytes; one without Wnt/β-catenin activation (CM1) and other with Wnt signaling activation (CM2). The expression of hepatospecific genes and the key regulator of hepatogenesis CEBP were achieved in both protocols. Similar differentiation results has been obtained by others authors using other stem cells [20].

Wnt/β-catenin pathway activation took place in CM2-treated cells, with nuclear β-catenin translocation and up-regulation of genes related to this pathway. Treatment of cells with another protocol (CM1) also induced hepatic differentiation but without the concurrence activation of Wnt/β-catenin pathway. We show for the first time the capability of CM1 (HGF+FGF7) to differentiate human MSC into hepatocytes. Our results show also that differentiation into hepatocytes may be induced with or without activation of Wnt/β-catenin pathway. Our results with CM1-treated cells are consistent with other studies where down-regulation of Wnt/β-catenin pathway during hepatic differentiation is observed [6], [7], [8]. On the other hand, in protocol CM2, dexamethasone was administered, and the administration of high dose of this glucocorticoid may be responsible of the nuclear β-catenin translocation observed. Others authors have demonstrated that equal concentration of dexamethasone induced osteogenesis of murine MSC via nuclear β-catenin translocation [15]. These data suggest that the down-regulation of this pathway is not essential for the differentiation of human MSC into hepatocytes. Therefore, in our hands the activation or inhibition of Wnt/β-catenin signaling pathway did not lead the hepatogenesis of human MSCs. However, the activation of Wnt signaling during hepatocytes differentiation might be associated with the generation of a tumoral phenotype and the expression of proteins related to liver cancer. Wnt/β-catenin pathway has been involved in the development, maintenance and differentiation of normal and malignant liver progenitor cells or MSC [16]. The sequence of molecular events leading to liver carcinogenesis is not well known. The accumulation of genetic alterations driving a cirrhotic liver to cancer is a multistep process originating from stem cells or mature hepatocytes [21]. Adult human MSC may be targets for malignant transformation and may undergo spontaneous transformation after long-term in vitro culture, supporting the hypothesis that some CSC originate from multipotential stem cells [22], [23]. *In vitro* data from transgenic mice suggest that activation of the Wnt/β-catenin pathway in epidermal stem cells leads to epithelial cancers [24]. The nuclear translocation of β-catenin in neoplastic hepatocytes leads to retrodifferentiation into immature hepatocyte progenitors [10]. Many *in vivo* studies have associated Wnt/β-catenin pathway activation with hepatic tumoral processes, such as hepatocellular carcinoma or hepatoblastoma [9], [25], [26]. Aberrant deregulation of Wnt signaling has been implicated as a major mechanism of liver tumorigenesis [27], [28] and up-regulation of Wnt signaling is a hallmark of hepatoblastoma, the predominant hepatic neoplasm in infants and children. Wnt/β-catenin activation has been found to be associated with increases in c-myc and cyclin D1 staining in tumours of patients with hepatoblastoma [29]. In the case of hepatocellular carcinoma molecular alterations responsible for its development and progression include: 1) loss of tumor suppressors genes, as p53 and/or activation of cyclin D1, 2) activation of oncoproteins as c-myc, and 3) alterations in Wnt signaling leading to nuclear accumulation of β-catenin [10], [30]. Our results show that the majority of these alterations (loss of p53, nuclear accumulation of β-catenin or c-myc overexpression), are present, along with the activation of Wnt signaling, in the hepatocytes obtained after CM2 treatment. Protein p53 is implicated in the control of cell cycle, apoptosis, DNA repair and angiogenesis and deregulation of p53 favors the development of liver tumor [31]. The loss of p53 has been described in many types of human tumors, particularly in 30%–60% of hepatocelular carcinoma contributing with the tumor progression [32]. The increases of c-*myc* observed in hepatocytes obtained by CM2 and the Wnt/β-catenin activation could also suggest a transformation of these cells into CSC. This hypothesis is reinforced with data obtained in CM2-treated cells related to an abnormal proliferation, higher PCNA expression, cell cycle alteration and secondary spheroids formation. These results suggest that in contrast to undifferentiated or CM1-treated cells, CM2-treated cells conserve stemness capability. This capability to form spheroids is intrinsic of stem cells or CSC. Sphere forming ability is known to be one of properties of CSCs [33], [34]. Secondary spheres formation after seeding cells at clonal density confirms that spheres formation reflects auto-renewal rather than cell aggregation. In addition the increased expression of CD13, CD49e, CD133, CD166 or VEGFR2 in CM2-treated cells suggests also similarities to CSC. Some proteins as CD13 or CD49e participate in process of chemotaxis, invasion and metastasis of malignant cells [35]. CD13 is an aminopeptidase N with matrix metalloproteinase activity that has been shown to play a role in tumor angiogenesis, invasion and metastasis, radiation resistance, and antiapoptosis [36], [37] and it has been involved with human liver CSC [38]. Haraguchi et al showed that the suppression of CD13 inhibited self renewal and the tumor initiation ability of CD13^+^cells [38]. CD49e, also known as integrin α5, is identified as one of the fibronectin receptor and its expression is increased in the hepatocellular carcinoma cell lines MHCC97 [39] and SMMC-7721 [35]. Angiogenesis is important for tumor growth, and is regulated by vascular endothelial growth factor (VEGF). Hepatocellular carcinoma is a solid tumor with rich neovasculature and VEGFR2 overexpression has been localized in tumoral hepatocytes [40]. CD133 is a CSC marker associated with radioresistance and chemoresistance in various cancers and has been also identified as specific antigenic marker of liver CSC [41], [42].

Finally, our proteomic analysis showed a higher presence of hepatocellular carcinoma-related proteins, such as cathepsin β precursor, cathepsin D precursor, adenine phosphoribosyl transferase, L-lactate dehydrogenase, triosephosphate isomerase, inorganic pyrophosphatase or peptidyl prolyl cis-trans isomerase, in CM2 treated cells compared to CM1 treated cells. A high expression of these proteins has been observed in hepatic tumor and metastasis [43], [44], [45]. Some of the detected proteins in this study participate in processes associated with the pathogenesis and the metastatic spread of hepatocellular carcinoma, such as cell motility and invasion, metabolism and signal transduction. Cathepsin-D has been reported to play an essential role in multiple tumor progression steps, affecting cell proliferation, angiogenesis, and apoptosis. Other reports also suggest that cathepsin D is a key mediator in induced apoptosis [46], [47]. Adenine phosphoribosyltransferase is an enzyme involved in the purine nucleotide salvage pathway, which is up-regulated in hepatocellular carcinoma and has been associated with Wnt/β-catenin activation [30]. Tumor formation is generally linked to increased activity of glycolytic enzymes, such as lactate dehydrogenase β [45], [43], [48] or triosephosphate isomerase [45], [49] and both proteins have been shown to be increased in CM2 treated cells in the present study. The reduction in LDH activity has been reported to result in diminished tumorigenicity, demonstrating that LDH plays a key role in tumor maintenance [50]. Peptidyl-prolyl isomerase (Cyclophilin A) has been implicated in several pathological processes, including hepatocellular carcinoma [51], [52]. Other studies have also showed the up-regulation of inorganic pyrophosphatase during hepatocellular carcinoma [45].

In contrast, other proteins are down-regulated in tumoral processes, including hepatocellular carcinoma. Tropomyosin β chain, transgelin or annexin A5, with a lower expression in CM2- treated cells compared to CM1-treated cells, are down-regulated proteins in hepatocellular carcinoma. Tropomyosin plays a role of stabilization of actin filaments and in the suppression of cellular transformation in non muscle cells, such as hepatocytes [53]. Other studies showed a decreased expression of this protein in hepatocellular carcinoma [54]. Transgelin is also a specific protein of smooth muscle cells, but its involvement in tumoral processes as a novel tumor suppressor protein has been documented. The loss of transgelin is a characteristic signature of colon and prostate carcinogenesis and its restoration suppresses colon tumorigenity in vivo and in vitro [55]. Besides, the promoter regions of transgelin are highly methylated in hepatocellular carcinoma [56]. Our study shows that the expression of transgelin was significantly decreased in CM2 vs. CM1 treated cells. Another protein with altered expression in our study is annexin A5. Annexins belong to a family of calcium-regulated phospholipid-binding proteins that has various intra- and extracellular roles in a range of cellular processes such as cell signalling, ion transport, cell division, and apoptosis [57]. The expression of DnaJ homologous subfamily B (member 11) was also decreased in CM2 vs. CM1 treated cells, and the decrease of this anti apoptotic protein may participate in the observed tumoral phenotype of CM2-treated cells.

In summary, our study demonstrates that Wnt/β-catenin down-regulation is not necessary for hepatocyte differentiation of MSC. We show for the first time a cross-talk between human bone marrow MSC hepatocytes differentiation, Wnt/β-catenin pathway and a tumoral phenotype. The activation of Wnt/β-catenin during human MSC differentiation into hepatocytes is associated with abnormal proliferation, expression of CSC markers, spheroid formation and the generation of liver cells with tumoral characteristics, in contrast to hepatocytes differentiated without Wnt/β-catenin activation. Exploration of the differences between cancer stem cells from normal stem cells is crucial not only for the understanding of tumor biology but also for the prevention of potential complications derived from future liver therapies with human MSC.

## Materials and Methods

### Ethics Statement

This study was approved by the Reina Sofia University Hospital Review Board. The procedures followed were in accordance with the ethical standards of the ethic committee from Hospital Reina Sofía and with the Declaration of Helsinki. All samples were collected after written informed consent.

### Human mesenchymal stem cells (MSCs) isolation

Human bone marrow (BM) was aspirated from the iliac crest of healthy donors. Fresh BM was cultured in flasks (FalconTM, BD Pharmigen, Franklin Lakes, NJ) seeding 10 µl BM cells/cm^2^ with alpha-minimum essential medium (α-MEM) supplemented with 2 mM L-glutamine, 15% fetal bovine serum (FBS) (BioWhittaker, Switzerland), 100 U/ml Penicillin, 0.1 mg/ml Streptomycin and 1 ng/ml of fibroblast growth factor-basic (FGF-b, Peprotech EC, London, UK) [58]. Cells were allowed to adhere for 48 h and non-adherent cells were washed out with phosphate-buffer saline (PBS) 100 mM pH 7,4 (Sigma-Aldrich, St Louis, MO). After 48 h, α-MEM supplemented with 10% FBS and 1 ng/ml FGF-b was added twice weekly. All cultures were maintained at 37°C in a humidified atmosphere containing 5% CO2. When adherent cells reached 90% confluence they were detached with 0.25% trypsin-EDTA (BioWhittaker, Switzerland), washed twice with PBS, centrifuged at 1800 rpm, 5 minutes and 4°C and replated in 6-well plates (SPL life sciences, Korea) at 10^3^ cells/cm^2^ and cultured under the same conditions.

### In vitro hepatic differentiation

Two different differentiation protocols were applied to confluent human MSCs for their differentiation into hepatocytes. An explicative diagram is included in Supporting Information (Figure S2). In the first protocol (conditioned medium 1, CM1), cells were cultured in α-MEM containing 10% FBS, 20 ng/ml hepatocyte growth factor (HGF) and 10 ng/ml fibroblast growth factor-7 for 21 days. The second protocol (conditioned medium 2, CM2) is based in the article by Kuan der Lee [17]. Briefly, human MSCs were previously treated with epidermal growth factor (EGF) 20 ng/ml and FGF-b 10 ng/ml for 48 h. Then, 20 ng/ml HGF, 10 ng/ml FGF-b and 0.61 g/L nicotinamide were added for one week. Finally, cells were treated for fourteen days with 1 µM dexamethasone, 20 ng/ml oncostatin and 10 µl/ml ITS. Treatments were refreshed 2–3 times per week. All cytokines were purchased from Peprotech EC (Paris, France), nicotinamide and dexamethasone were obtained from Sigma-Aldrich (St Louis, MO) and ITS from BD Pharmigen (Franklin Lakes, NJ, USA). Nuclear and cytoplasmic proteins and RNA were collected at 7, 14 and 21 days of culture.

### Flow Cytometry Analysis

For immunophenotype studies, basal and differentiated human MSC were detached and stained with fluorescein- or phycoerythrin-coupled antibodies and analyzed with a FACSCalibur flow cytometer (Becton, Dickinson). Anti-CD34-FITC, anti-CD45-PE and anti-CD133 were purchased from Miltenyi Biotec (Berlin, Germany), anti-CD73-PE, anti-CD90- PE and anti-CD166 were from BD Pharmigen (Franklin Lakes, NJ), anti-CD13-FITC, anti-CD44-FITC and anti-CD49e-FITC were from Beckman Coulter, Inc (CA, USA), anti-CD105-FITC was from R&D Systems (MN, USA), and anti-CD29-FITC, anti-CD184-PE and VEGFR2 were from eBioscience, Ltd (London, UK).

### Quantitative real time RT- PCR analysis

Total RNA was extracted following a modification of Chomezynski and Sacchi's protocol with Trizol reagent Sigma-Aldrich (St Louis, MO). Total RNA was quantified by spectrophotometry (ND-1000, Nanodrop Tecnologies, DE, USA). One µg of total RNA was treated with DNAse (DNAse kit, Sigma-Aldrich, St Louis, MO) and complementary DNA was amplified using the QuantiTect Reverse Transcripction kit (Qiagen, Hilden, Germany). Primers were designated with the free Oligo 7 software and their sequences are listed in Table 3. Quantitative real-time PCR was performed in a Light cycler 480 (Roche Diagnostics, Basel, Switzerland).

**Table 3:** Primers used for quantitative RT-PCR analyses.

**Albumin (ALB)**	**F:**	5′ TGA GAA AAC GCC AGT AAG TGA C 3′
	**R:**	5′ TGC GAA ATC ATC CAT AAC AGC 3′
**α-Fetoprotein** **(AFP)**	**F:**	5′ GCT TGG TGG TGG ATG AAA CA 3′
	**R:**	5′ TCC TCT GTT ATT TGT GGC TTT TG 3′
**CK18** **(KRT18)**	**F:**	5′ CCC GTC ACG CCC TAC AGA T 3′
	**R:**	5′ ACC ACT TTG CCA TCC ACT ATC C 3′
**C/EBPα** **(CEBPG)**	**F:**	5′ CCC GCC CGT GGT GTT ATT 3′
	**R:**	5′ GGT TGC GTC AGT CCC GTG TA 3′
**Cytochrome P450** **CYP3A5**	**F:**	5′ GAT CCC CTT GAA ATT AGA CAC G 3′
	**R:**	5′ TTG AAA TCT CTG GTG TTC TGG 3′
**α1-antitrypsin** **(SERPINA1)**	**F:**	5′ AAG GTG CCT ATG ATG AAG CGT 3′
	**R:**	5′ GTG ATG CCC AGT TGA CCC A 3′
**Lrp5/6**	**F:**	5′ GCA GCC TTT CTT CCA CAC TC 3′
	**R:**	5′ CTC CTG CCT TAC ACG TCC T 3′
**Frizzled-3** **(FZD3)**	**F:**	5′ TGG AGC CAT TCC ACC CTA TG 3′
	**R:**	5′ GAA CCT ACT GCA TTC CAT ATC 3′
**c-** ***myc*** **MYC**	**F:**	5′ ACC ACC AGC AGC GAC TCT GAG GA 3′
	**R:**	5′ CGT AGT TGT GCT GAT GTG TGG AGA 3′
**18S Ribosomal** **RN18S1**	**F:**	5′ GTA ACC CGT TGA ACC CCA TT 3′
	**R:**	5′ CCA TCC AAT CGG TAG TAG CG 3′

### Immunocytochemical analysis

Human MSCs were cultured on chamber slides (Nunc, Rochester, NY, USA) for 21 days and then were fixed and treated during 20 min with 0.01 M citrate buffer pH 6. Cells were incubated for 1 h at room temperature with: anti-PCNA (1∶75 dilution, Santa Cruz Biotechnologies, Santa Cruz, CA, USA), anti-albumin (DakoCytomation Glostrup, Denmark, 1∶2000 dilution), anti-α-fetoprotein (R&D Systems, Minneapolis, MN, USA, 10 µg/ml), anti-cytokeratin-19 (R&D Systems, Minneapolis, MN, USA, 10 µg/ml), or anti-α-1-antitrypsin (DakoCytomation Glostrup, Denmark, 1∶800 dilution) primary antibodies. HRP-labelled polymer conjugated to secondary antibodies was used for 30 minutes at room temperature and diaminobenzidine was added to detect positive staining. Finally, cells were counterstained with hematoxylin (DakoCytomation Glostrup, Denmark). During all the procedure three washes with PBS were performed after each step.

### Confocal microscopy analysis

Undifferentiated and differentiated human MSCs were cultured on chamber slides and, after the corresponding treatments; they were fixed with 4% paraformaldehyde (Sigma-Aldrich) for 15 minutes at room temperature. Samples were then treated with chilled methanol (−20°C) for 10 min and washed in PBS (3×, for 5 min) and then sequentially incubated for 60 minutes each with anti-β-catenin (1∶50, BD Pharmigen, Franklin Lakes, NJ) and anti-mouse IgG-FITC (DakoCytomation Glostrup, Denmark). Between incubations, slides were washed with PBS+1% BSA (Sigma-Aldrich) for 10 minutes. DAPI (Invitrogen, CA, USA) was used for nuclear stain. Cells were examined by confocal fluorescence microscopy using a confocal microscope (LSM 5 Exciter Carl Zeiss).

### Cell count

The number of undifferentiated cells, CM1 and CM2-treated cells was counted at 0, 7, 14 and 21 days of culture. Cells were treated with Trypsin-EDTA (Sigma), inactivated with medium plus FBS and washed with PBS. Trypan blue was used to measure the cellular viability and the count was carried out with a Neubauer chamber.

### Cell cycle

For cell cycle, the different types of cells (undifferentiated, CM1 and CM2-treated cells) were harvested after 21 days of differentiation. Cells were trypsinized and subsequently fixed in 70% cold ethanol overnight. After cells were centrifuged and washed with Hank's solution 1× (Sigma-Aldrich, St Louis, MO) twice. Cells were lysated with DNA extraction buffer which contained citric acid 0.1 M and anhydrous disodium phosphate 0.2 M (Sigma-Aldrich, St Louis, MO) for 5 minutes. After incubation, cells pellets were resuspended in 100 µl staining buffer which contining 50 µg/ml propidium iodine, 50 µg/ml RNase, 0.1% Triton-X-100 and 0.1 M EDTA in PBS (Sigma-Aldrich, St Louis, MO). Cells were incubated for 30 min in darkness. Finally, cells were resuspended in PBS and they were acquired at low speed using FACScaliber (Becton Dickinson, CA, USA). Cell cycle analysis was performed on FlowJo program based on the mathematical algorithm of Watson (Becton Dickinson, CA, USA).

### Spheroid formation assay

Figure S3 from Supporting Information section indicates the followed steps for spheroids assay. After 21 days of culture, cells were collected with Trypsin-EDTA and harvested at 50000 cell/ml in low adherence plates (6 wells) with DMEM:H12 medium without glutamine, antibiotics or serum and plus 20 ng/ml EGF (Peprotech, NJ, USA), 10 ng/ml bFGF (Peprotech), B27 1× (Invitrogen, CA, USA) and insulin 100 IU (Novo Nordisk, Bagsvaerd, Denmark). After 4 days of culture, spheres formation was visualized in a microscope and the number of cells after trypsin- EDTA digestion was counted.

To analyze the number of secondary spheroids undifferentiated cells, CM1 and CM2 treated cells were harvested at clonal dilution (cell/ul) on low adherence plates. After 4 days of culture the number of spheres was counted in an inverted microscope. Three experiments were carried out and the data are expressed as mean of number of spheres ± standard deviation. Representative microphotographs of secondary spheroids were taken in an inverted microscope to 10×.

To check 3-dimensional structure of spheroids, undifferentiated cells, CM1 and CM2-treated cells were collected from plates and stained with DAPI for 5 minutes. Subsequently cell were centrifuged gentlely and resuspended in 15 ul of PBS. Spheroids' mounting was carried out according to the protocol described by Weiswald et al [59]. For 3D reconstruction, a stack of confocal images was collected through the spheroids with step size of 0.488 µm between adjacent optical planes, starting from one pole of the spheroids. 360° 3D projects plugging from ImageJ was used to generate a 3D animation.

### Two-dimensional difference gel electrophoresis (2D-DIGE) analysis

After acetone precipitation, protein samples (Control cells at 0 and 21 days of culture and hepatocytes obtained by CM1 or CM2) were solubilized in 2-D DIGE sample buffer: 7 M urea, 2 M thiourea, 4% CHAPS, 30 mM Tris, buffered to pH 8. Protein concentration was determined using the Bradford's assay (Bio-Rad). Then, 50 µg protein was labelled with 400 pmol of CyDye DIGE Fluor minimal dyes (GE Healthcare) and incubated on ice in the dark for 30 min according to the manufacturer's instructions (Cy3, Cy5 for samples and Cy2 for internal control consisting of a mixture composed by equal amounts of protein from all samples). Paired samples were reverse-labeled in order to prevent potential dye labeling bias. The reaction was stopped by addition of 1 µl of 10 mM lysine and incubated on ice for 10 min. Samples were cup-loaded onto IPG strips, 24 cm, pH 3–11NL (GE Healthcare), and subjected to isoelectrofocusing (IEF) in IPGphor™ IEF System (GE Healthcare) according to the manufacturer's recommendations. Upon IEF, strips were incubated in equilibration buffer (50 mM Tris-HCl, pH 8.8, 6 M urea, 30% glycerol, 2% SDS, a trace of bromophenol blue), containing 0.5% DTT for 15 min and thereafter in the same buffer with 4.5% iodoacetamide for 15 min. For the second dimension, strips were loaded on top of 12.5% polyacrylamide gels and run (1 W/gel) for 12–14 h until the bromophenol blue dye reached the gel bottom-end. Subsequently, 2D gels were scanned using a TyphoonTM Trio Imager (GE Healthcare) at 100 µm resolution with λex/λem of 488/520, 532/580, and 633/670 nm for Cy2, Cy3, and Cy5 respectively. The photomultiplier tube was set to ensure that the maximum pixel intensity was between 90,000 and 99,000 pixels. Image analysis was performed using DeCyder 6.5 software (GE Healthcare) as described in the user's manual. Three independent experiments were performed for each experimental setup. Briefly, the differential in-gel analysis module was used for spot detection, spot volume quantification and volume ratio normalization of different samples in the same gel. Then the Biological Variation Analysis (BVA) module was used to match protein spots among different gels and to identify protein spots that exhibit significant differences. Manual editing was performed in the BVA module to ensure that spots were correctly matched between different gels, and to get rid of streaks and speckles. Differential expressed spots were considered for MS analysis when the fold change was larger than 1.2 and the p-value after T-test was below 0.05. Preparative gels were run with 350 µg of protein following the same procedure described above. Proteins were visualized by staining with SYPRO Ruby Protein Gel Stain (Bio-Rad) and images were acquired with a TyphoonTM Trio Imager using λex/λem of 532/560 nm. Spots differentially represented were excised manually and gel specimens were processed with a MassPrep station (Waters). In-gel tryptic digestion was performed with 12.5 ng/µl trypsin in 50 mM ammonium bicarbonate for 12 h at 37°C. The resulting peptides were extracted with 5% formic acid, 50% acetonitrile. Samples were then concentrated in a speed-vac before MS analysis.

### Protein identification by LC-ESI-MS/MS analysis

Microcapillary reversed phase LC was performed with a CapLCTM (Waters) capillary system. Reversed phase separation of tryptic digests was performed with an Atlantis, C18, 3 µm, 75 µm×10 cm Nano EaseTM fused silica capillary column (Waters) equilibrated in 5% acetonitrile, 0.2% formic acid. After injection of 6 µl of sample, the column was washed during 5 min with the same buffer and the peptides were eluted using a linear gradient of 5–50% acetonitrile in 30 min at a constant flow rate of 0.2 µl/min. The column was coupled online to a Q-TOF Micro (Waters) using a PicoTip nanospray ionization source (Waters). The heated capillary temperature was 80°C and the spray voltage was 1.8–2.2 kV. MS/MS data were collected in an automated data-dependent mode. The three most intense ions in each survey scan were sequentially fragmented by CID using an isolation width of 2.5 and relative collision energy of 35%. Data processing was performed with MassLynx 4.0. Database searching was done with ProteinLynx Global Server 2.1 (Waters) and Phenyx 2.2 (GeneBio, Geneva, Switzerland) against Uniprot knowledgebase Release 12.3 consisting of UniprotKB/Swiss-Prot Release 54.3 and UniprotKB/TrEMBL Release 37.3 with 285.335 and 4.932.421 entries respectively. The search was enzymatically constrained for trypsin and allowed for one missed cleavage site. Further search parameters were as follows: no restriction on molecular weight and isoelectric point; fixed modification, carbamidomethylation of cysteine; variable modification, oxidation of methionine.

### Preparation of cell lysates for Western Blot

Cytosolic extracts were obtained with a lysis buffer A, pH 7.9, containing 10 mM Hepes, 10 mM KCl, 0.1 mM EDTA, 0.1 mM EGTA, 1 mM DTT, 0.5 mM PMSF, 70 µg/ml Protease Inhibitor Cocktail, 0.5% Igepal CA-630 (Sigma-Aldrich, St Louis, MO). The suspension was centrifuged (13000 rpm, 3 min and 4°C) and supernatant was stored at −80°C until used. Nuclear extracts were obtained by incubating the pellet obtained as described above in a lysis buffer B, pH7.9, containing 20 mM Hepes, 0.4 mM NaCl, 1 mM EDTA, 1 mM EGTA, 1 mM DTT, 1 mM PMSF, 46 µg/ml Protease Inhibitor Cocktail (Sigma-Aldrich, St Louis, MO). Protein concentration was determined using the Bradford assay (Bio-Rad Laboratories GmbH, Munich, Germany). For Western Blot analyses, equal amounts of protein were loaded and electrophoresed on 7% SDS-polyacrylamide gel (Invitrogen; CA, USA). The protein was transferred to a nitrocellulose membrane (Invitrogen; CA, USA), and blots were incubated in blocking solution (Bio-Rad Laboratories GmbH, Munich, Germany). Primary antibodies were diluted in TTBS+5% non fat dry milk powder. Anti-β-catenin antibody (Cell Signaling, Boston, MA, USA) was diluted 1∶1000 before use and anti-TFIIB (1∶500 dilution, Santa Cruz Biotechnology) was used as loading control of nuclear extract. Other primary antibodies used were: anti-p53 (1∶500) and anti-L-lactate dehydrogenase β chain (1∶200) from Santa Cruz Biotechnologies, anti-tropomyosin β chain (1∶500), adenine phosphoribosyltransferase (1∶500) and Transgelin (1∶2000) that were purchased from Novus Biologicals Littleton, CO, cathepsin B (4 µg/ml) from Sigma-Aldrich and tubulin 1∶10000 from Abcam (Cambridge, UK) were performed. Blots were immunolabeled using a horseradish peroxidase conjugated secondary antibody and developed on autoradiographic film using the ECL Plus Western Blotting Detection System from Amersham Biosciences U.K. Limited (Little Chalfont, England).

### Statistical analysis

Data are expressed as mean ± SD. The difference between means from two different groups was evaluated by performing a t test and *p* values less than 0.05 were considered significant. The data analysis was performed with SPSS.11 software.

## Supporting Information

Figure S1 **Movie showing the 3D structure of a representative spheroid in CM2-treated cells.**
(AVI)

Figure S2 **Explicative diagram of both differentiation protocols (CM1 and CM2).**
(TIF)

Figure S3 **Explicative diagram of spheroid formation assay.**
(TIF)
