# Supplementary figures and images for: Nuclear Translocation of β-Catenin during Mesenchymal Stem Cells Differentiation into Hepatocytes Is Associated with a Tumoral Phenotype
Source: PLoS One. 2012 Apr 10;7(4):e34656. doi: 10.1371/journal.pone.0034656 (PMC3323576; doi:10.1371/journal.pone.0034656)

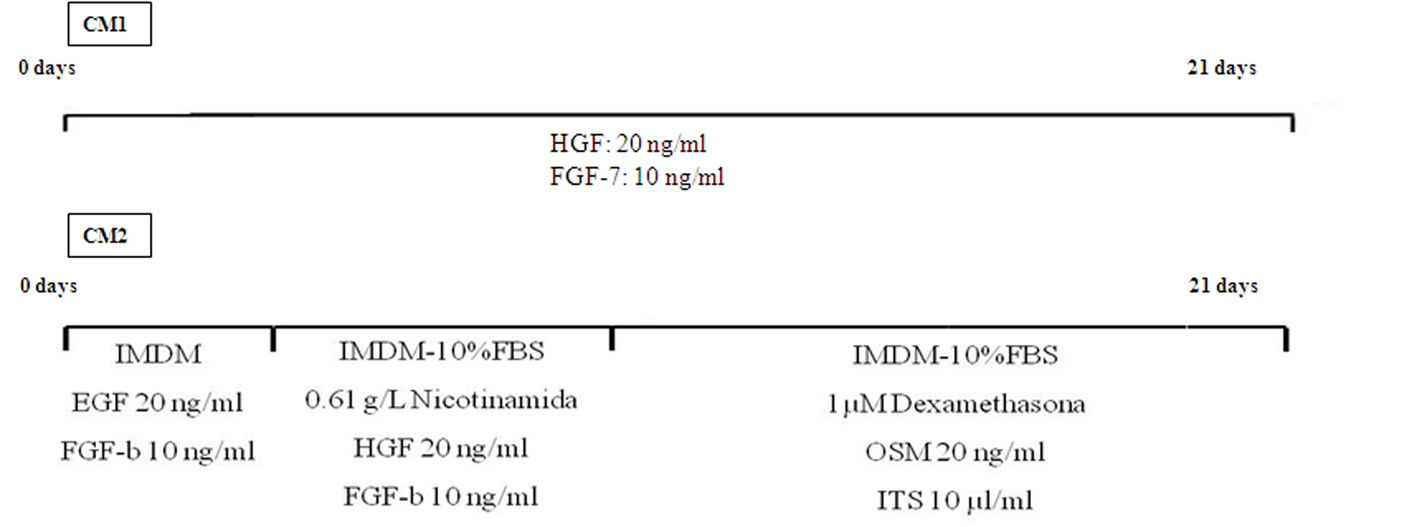

Supplement: Figure S2 — Explicative diagram of both differentiation protocols (CM1 and CM2). (TIF) [file pone.0034656.s002.tif]

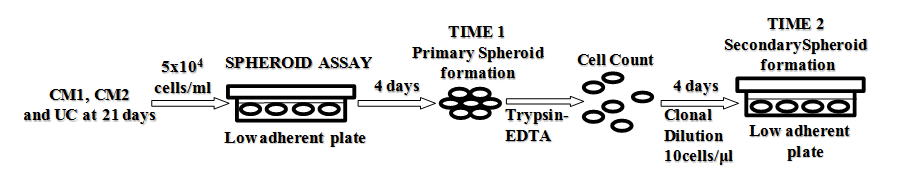

Supplement: Figure S3 — Explicative diagram of spheroid formation assay. (TIF) [file pone.0034656.s003.tif]
